# On Phase Identification of Hardened Cement Pastes by Combined Nanoindentation and Mercury Intrusion Method

**DOI:** 10.3390/ma14123349

**Published:** 2021-06-17

**Authors:** Jingwei Ying, Xiangxin Zhang, Zhijun Jiang, Yijie Huang

**Affiliations:** 1 School of Civil Engineering and Architecture, Guangxi University, Nanning 530004, China; xianz184@163.com (X.Z.); 17375680026@163.com (Z.J.); 2 Key Laboratory of Engineering Disaster Prevention and Structural Safety of Ministry of Education, Guangxi University, Nanning 530004, China; 302huangyijie@163.com; 3 Guangxi Key Laboratory of Disaster Prevention and Engineering Safety, Guangxi University, Nanning 530004, China; 4 Shandong Key Laboratory of Civil Engineering Disaster Prevention and Mitigation, Shandong University of Science and Technology, Qingdao 266590, China

**Keywords:** nanoindentation, pore structure, cement paste, cluster analysis, deconvolution analysis

## Abstract

The micro-mechanical properties of hardened cement paste can be obtained by nanoindentation. Phases at different locations can generally be determined by using the Gaussian mixture model (GMM) method and the K-means clustering (KM) method. However, there are differences between analysis methods. In this study, pore structure and porosity of hardened cement paste aged three, seven, and 28 days were obtained by mercury intrusion porosimetry (MIP), and their micro-mechanical properties were obtained by the nanoindentation method. A new method, GMM-MIP and KM-MIP, was proposed to determine the phase of hardened cement paste based on the pore structure and nanoindentation results. The results show that GMM-MIP and KM-MIP methods are more reasonable than GMM and KM methods in determining the phase of hardened cement paste. GMM-MIP can be used to obtain reasonable phase distribution. If the micro-mechanical properties of each phase in hardened cement paste do not satisfy the normal distribution, the GMM method has significant defects.

## 1. Introduction

Climate change is one of the most critical issues of our time. However, there is still a long way to go to achieve the goal of the Paris Agreement. Like all energy and carbon dioxide intensive industries, the global cement industry urgently needs to promote low-carbon transformation. Cement-based composites are widely used in civil engineering. Hardened cement pastes can be regarded as composites consisting of phases with different mechanical properties on both micro and nanoscale. The composition and micro-mechanical properties of hardened cement paste influence concrete macro-mechanical properties and durability [1,2]. Nanoindentation technology can be used to obtain the mechanical properties of cement clinker and the micromechanical properties of hydration products and porosity. For example, in order to study the macroscopic response of non-uniformly degraded hardened cement paste, Brow [1] used nanoindentation technology to obtain the elastic modulus of each indentation point and then used the gauss convolution method to identify each phase component of hardened cement paste. The microstructure and micromechanical properties of hardened cement paste have an important influence on concrete engineering. The development of various characterization techniques provides an important tool for understanding the microstructure and properties of materials [3], while these characterization methods need to be further developed [4].

At present, nanoindentation technology is widely used to study the micromechanical properties of cement. Due to the high heterogeneity of cement-based materials in multi-scale [5], it is difficult to test a specific phase repeatedly. Therefore, grid lattice (large array of nanoindentation) indentation testing technology is mainly used in the nanoindentation statistical analysis method. Němeček [6] proposed a new nanoindentation acceleration testing method to realize the calculation and analysis of each indentation point of cement-based materials micromechanical properties, but this method is only suitable for a small area (≈103 μm^2^). Statistical nanoindentation technology assumes that the mechanical properties of each indentation point are independent of each other, and the experimental data obey normal distribution. Then, the experimental data are convoluted by Gaussian fitting. After the data sets of different mechanical properties are obtained, the types of phases and the statistical characteristics of mechanical properties of each phase can be further obtained. Lee [2] investigated the creep properties of cement and alkali-activated fly ash paste and mortar determined from statistical analysis of nanoindentation data. Based on the principle of statistical nanoindentation, many scholars have carried out a series of research. Their research contents include microfracture, nano scratch and nanoindentation. In the field of microfracture, to investigate the mesoscopic fracture of heterogeneous cement-treated base materials, Zhao [7] regarded concrete as a non-uniform material composed of mortar, aggregate, pore, initial defect and interface transition zone, and established a meso model of concrete by using discrete element method and random aggregate method, and then analyzed the fracture performance of hardened cement paste. In order to study the fracture toughness of cement paste after hydration, Gautham [8] used the grid indentation method to analyze the micromechanical properties of hardened cement paste. That is, the nanoindentation point was 10 × 10, i.e., 100 indentation points. Then the phase of hardened cement paste was obtained by the Gauss convolution method. In order to study the micromechanical properties of hardened cement paste, Zhang [9] made a 100-micron cube by a precision cutting method. Then, the micro specimen was split by the tip of the indenter, and the Weibull distribution of the fracture strength was obtained. In the field of nano scratch, Liu [4] studied the hardness of hardened cement pastes at different scales by the cube-corner scratches method, and the results show that the scratch curve was sensitive to the hardness of the local phase. However, due to the relationship between pore phase and surface roughness, this scratch method was difficult to detect pore phase. In the field of nanoindentation, Akono [10] investigated the physical properties of cement with nano-TiO_2_ using depth-sensing-based methods such as statistical nanoindentation and microscopic scratch testing and statistical deconvolution show an increase in the fraction of high-density calcium silicate hydrates and calcium hydroxide.

The nanoindentation method has excellent advantages in characterizing the micromechanical properties of materials but has significant disadvantages in characterizing the microstructure of materials. Engineering cement-based composites require a comprehensive understanding of the microstructure features governing macroscopic properties. Wilson [11] fostered the latest chemo-mechanical technique to disclose the micro-mechanical properties of intimately intermixed phases. In order to determine the different microstructure characteristics and understand the formation mechanism of the hardened paste phase, Wei [12] used the quantitative modulus mapping coupling technology in the form of scanning probe microscope image, nanoindentation, SEM and so on. The chemical mechanical morphology of C–S–H gel in ordinary silicate cement and slag cement paste was studied by X-ray spectrometer. Roa [13] studied the correlation between the microstructure and the mechanical properties of the metallic cobalt binder in cemented carbide systems. In Roa’s study [13], multiple large indentation maps with 10,000 indents per map on a 50 × 50 μm^2^ area was performed to a maximum load of 4 mN. In addition, Li [3] reported modulus mapping applications, peak force quantitative nanomechanical mapping, and nano scratch for research on the micro and nanoscale compositions, structures, and mechanical properties of modem cement-based materials. To investigate the quantitative nanomechanical properties of the same indent location in hardened cement paste, Li [14] applied three types of nanomechanical methods, including static nanoindentation, modulus mapping and peak-force quantitative nanomechanical mapping. Furthermore, Rakowiak [15] presented a novel approach for the chemo-mechanical characterization of cement-based materials, which combines the classical grid indentation technique with elemental mapping by scanning electron microscopy-energy dispersive X-ray spectrometry.

Cluster analysis is one of the methods in the data processing. The characteristic of cluster analysis is to classify similar data into the same category. In this way, a large amount of data can be reasonably divided into different categories. The advantage of clustering analysis is that it will not be affected by the number of data, which is different from the method of using probability method and least square method to divide data types. Due to the algorithm’s flexibility, cluster analysis technology has been widely used in the data analysis of atmospheric science in recent 50 years [16]. K-means is one of the most commonly used clustering analysis techniques. For example, Konstantopoulos [17] performed data labelling with unsupervised machine learning with k-means clustering to test novel Portland cement formulations with Carbon Nanotubes and intrinsic properties revelation. Due to the problems of the presupposition of mechanical properties of materials, complicated calculation, and uncertainty of initial value selection in the Gauss convolution method, Hou [18] applied cluster analysis to the study of nanoindentation of cement-based materials, and Krakowiak [19] compared the differences of different clustering methods and explained the applicability of clustering analysis method, which can increase the stability of the results. It is known from the above analysis that nanoindentation is an effective means to characterize the micromechanical properties of hardened cement paste. Since statistical nanoindentation methods are not perfect, it is usually necessary to comprehensively characterize the properties of hardened cement paste in combination with other methods.

In order to divide the phase of hardened cement paste more accurately, the advantages and disadvantages of different methods need to be comprehensively compared. In this study, the surface roughness of the samples was measured by atomic force microscope, and the Micro-mechanical properties of hardened cement pastes aged three, seven, and 28 days were tested by a nanoindentation instrument. Their micro-pore structure was tested by a mercury intrusion porosimeter (MIP). A new method for dividing cement phases based on the test results of the MIP method and nanoindentation method was proposed. Based on the nanoindentation results of hardened cement pastes at different ages, the advantages and disadvantages of the K-means method and Gaussian mixture models method are comprehensively compared.

## 2. Materials and Fabrication Methods

The cement used was Ordinary Portland Cement with 42.5 MPa according to the Chinese national standard “cement mortar strength test method (ISO method)” GBT 17671-1999 (manufacturer: Guangxi Fusui Conch Cement Co., Ltd.; Origin: Guangxi Province, China). The cement composition is shown in Table 1.

**Figure 1:**
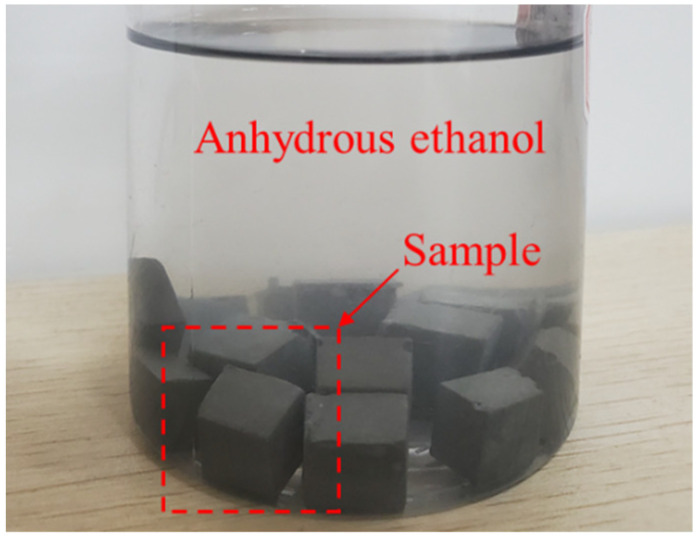
Sample soaked in anhydrous ethanol.

The water to binder ratio of hardened cement paste is 0.4. The sample fabrication method was as follows: drinking tap water and cement powder were placed together into a cement gel sand electric blender and stirred first at low speed (speed of 60 rpm) for 2 min and then at high speed (speed of 120 rpm) for 1 min. The cement paste was then poured inside an orthopaedic silicone test mould with a side length of 10 mm. They were then placed on a small shaking table and vibrated at high frequency (2860 beats per minute) for 30 s. A thin film cover was employed in order to prevent moisture evaporation, and the mould was removed after 24 h. Then, the samples were immersed in saturated Ca(OH)_2_ solution with room temperature controlled at 20 ± 1 °C. At 3, 7, and 28 days, portions of the sample were removed and placed inside a vacuum drying box. The samples were vacuum dried for 24 h with a drying temperature of 60 °C and a vacuum vessel pressure of 133 Pa. The vacuum drying box model is ZKXF, and the manufacturer is Shanghai Yiheng (Shanghai, China). Then, the dried specimens were immediately immersed in absolute ethanol to stop the cement’s hydration reaction, as shown in Figure 1.

**Figure 2:**
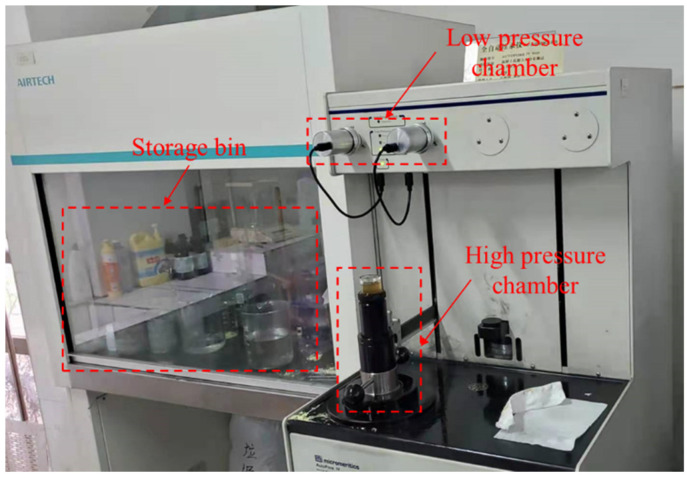
Pore structure test.

Before testing the pore structure of the samples, the test pieces were taken out from the absolute ethanol solution and re vacuum dried for four hours. For each kind of samples with the same mix proportion and the same curing age, they were tested three times respectively. The manufacturer of the automatic mercury porosimeter is Micromeritics company, and the model is AutoPore IV 9500. (Norcross, GA, USA) The mercury pressure gauge was used at a maximum pressure of 30,000 psi, and the pore size range was determined to be from 6 nm to 302 μm. The test process is shown in Figure 2.

Before nanoindentation testing, the sample vessel was evacuated at a vacuum of 2 Pa, and the sample was then soaked in an epoxy solution. After waiting for the epoxy to harden, the test blocks were ground sequentially. The particle size of the abrasive cloth used for grinding and the particle size of the polishing liquid are both coarse to fine. The particle size in the diamond polishing solution was 3 µm, 1 µm, 0.25 µm, and 0.05 µm. Each polishing solution was fitted with a separate polishing cloth. The polishing time for each polishing solution was between 1 h and 2 h.

**Figure 3:**
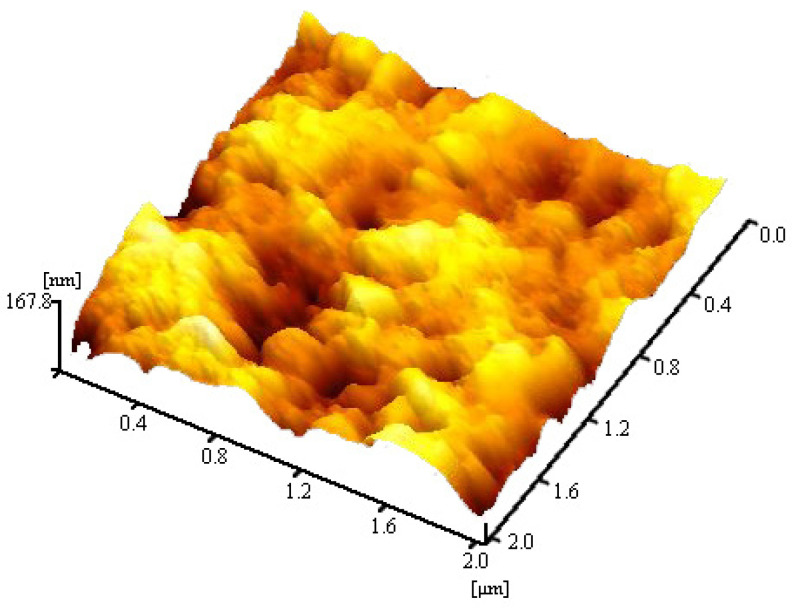
Scanning results of an atomic force microscope.

The surface roughness of samples affects the accuracy of nanoindentation test results [20,21]. Atomic force microscope (model: 5100n, manufacturer: Hitachi, Tokyo, Japan) was used to scan the sample surface. The samples are hardened cement pastes with a curing age of 28 days and water binder ratio of 0.4. The result of scanning is shown in Figure 3.

It can be seen from the figure that the height difference of the sample surface is within 168 nm, which meets the requirements of test accuracy.

**Figure 4:**
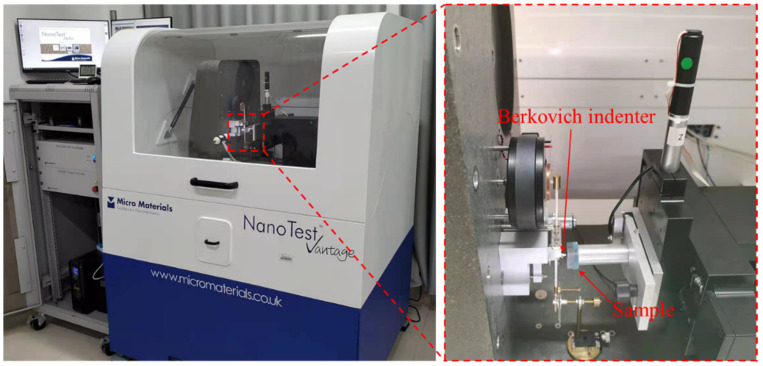
Nanoindentation testing.

The load–displacement curve of the sample surface was obtained by nanoindentation. The model of the indentation instrument is a nano test. The manufacturer of the indentation instrument is Micro Materials Ltd. (Wrexham, UK), as shown in Figure 4.

**Figure 5:**
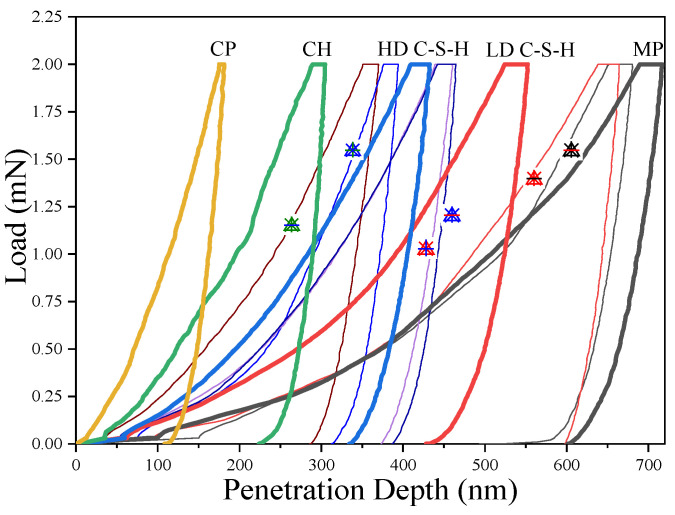
Typical load-depth curve of each phase at indentation point.

The number of indentation points in each region is 20 rows and 20 columns, and the distance between indentation points is 20 microns. After the indenter touched the sample’s surface, it was loaded to 2 mN at a rate of 0.2 mN/s. Then, the load of 2mN is maintained for 5 s to eliminate the effect of creep. The unloading speed is the same as the loading speed. The physical phases of the hardened cement paste include the unhydrated cement particles (CP), the calcium hydroxide crystal (CH), the high-density calcium silicate hydrate (HD C–S–H), the low-density calcium silicate hydrate (LD C–S–H), and the pore phase (MP) method [5]. A typical load–displacement curve is shown in Figure 5.

The reference method [22] is used to calculate the elastic modulus (E) and hardness (H) of hardened cement paste at the indentation point. The calculation formula is shown in formula (1) and (2),

(1) $$\frac{1}{E_{r}}=\frac{1-v_{s}^{2}}{E}+\frac{1-v_{i}^{2}}{E_{i}}$$

(2) $$H=P_{max}/A$$

where *E_r_* is the elastic modulus of the sample, *E_i_* is the elastic modulus of the indenter, and *E_i_* is 1141 GPa; *v_i_* is the pressure head Poisson’s ratio, and the value is 0.07; vs. is the Poisson’s ratio of cement-based materials, and the value is 0.2. A is the contact area and *P_max_* is the maximum load.

## 3. Analysis of Test Results

### 3.1. Porosity and Pore Structure

**Figure 6:**
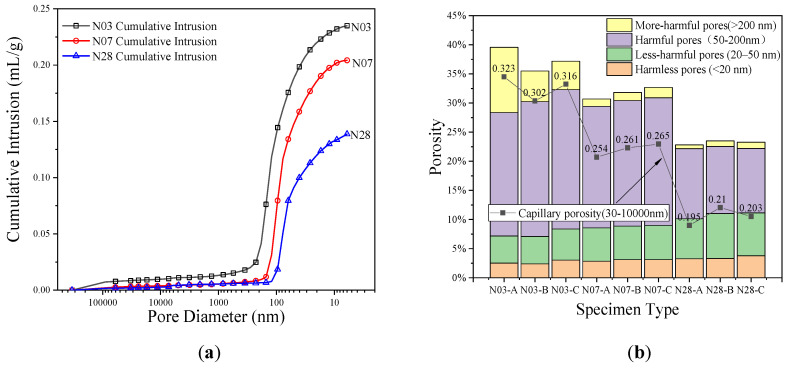
Pore structure test results for samples of all ages: (**a**) accumulated mercury content changes with pore size. (**b**) different types of porosity.

The pore structure of hardened cement paste obtained by the mercury intrusion porosimetry (MIP) is shown in Figure 6.

In this figure, N03, N07, and N28 correspond to hardened cement pastes aged three, seven, and 28 days respectively. It can be seen from Figure 6a that, for the same pore size, the cumulative mercury uptake curve decreased significantly with the increase of curing time. For example, compared with three-day-old hardened cement paste, the cumulative mercury uptake curves of the seven-day-old and 28-day old cement paste at a pore size of 10 nm decreased by 13.12% and 42.54%, respectively. Compared with three-day-old cement paste, the most probable pore size of seven-day-old cement paste and 28-day old cement paste decreased by 21.00% and 36.17%, respectively. This shows that the pore structure of hardened cement paste is gradually refined with the increase of curing time.

According to the influence of pore size on the durability of concrete, the porosity can be divided into four categories [23]: harmless pore (<20 nm), less-harmful pore (20–50 nm), harmful pore (50–200 nm), and more harmful pore (>200 nm). The pore size from 30 nanometers to 1000 nanometers is defined as the capillary adsorption pore [24,25]. Porosities of different classes are calculated from the mercury ingress curve and the density of the sample, as shown in Figure 6b. It can be seen from the figure that as the age increases, the harmful pores in hardened cement paste gradually decrease. For example, compared with the three-day age, seven-day age and 28-day age-hardened cement paste reduced 73.45% and 88.23%, respectively for more harmful pores. For harmful pores, the reduction was 6.83% and 47.89%, respectively. However, for less harmful pores, the increase was 22.49% and 4.58%, respectively. The reduction was 29.88% and 36.27% for harmless pores, respectively, and 10.38% and 33.82% for the total porosity. For the capillary pore, the reduction was 13.34% and 35.36%, respectively.

### 3.2. Comparison of Different Methods of Phase Identification

The elastic modulus and hardness obtained by nanoindentation consist of a variety of phases. Generally, mathematical methods for determining different phases include GMM and K-means clustering. For GMM, it is assumed that the modulus of elasticity and hardness of each phase follows a normal distribution [26]. The deconvolution calculation of GMM models is performed by using the minimum square difference method, in which several variables can be considered simultaneously. The principle of the K-means method [19] is to perform a cluster analysis based on both moduli of elasticity and hardness.

Porosity in cement corresponds to a lower modulus of elasticity and hardness. Assuming that the proportion of indentation points of the pore to the total indentation points is equal to the porosity, the indentation point data corresponding to the pore can be excluded. Then, the above two analytical methods are carried out. These two methods are called GMM-MIP and K-Means-MIP, respectively. Specific steps are as follows: First, the modulus of elasticity and hardness values at each indentation point is normalized as shown in Equations (3)–(5). All $\delta_{i}$ in Equation (5) are split into two parts. The minor part accounts for the same proportion of all data points as the porosity obtained by the mercury intrusion method. The minor part corresponds to the pore phase. Then, GMM and K-Means are used to analyze the rest.

(3) $$h_{i}=\frac{H_{i}-\min\left(H\right)}{\max\left(H\right)-\min\left(H\right)}$$

(4) $$e_{i}=\frac{E_{i}-\min\left(E\right)}{\min\left(E\right)-\max\left(E\right)}$$

(5) $$\delta_{i}=\sqrt{h^{2}+e^{2}}$$

where min (*H*) and Max (*H*) represent the maximum and minimum hardness values for all hardness values, Min (*E*) and Max (*E*) represent the maximum and minimum values of all Young’s modulus values. *H_i_* and *E_i_* represent the 1st hardness and elastic modulus. The *δ_i_* represents the distance from the original point.

**Figure 7:**
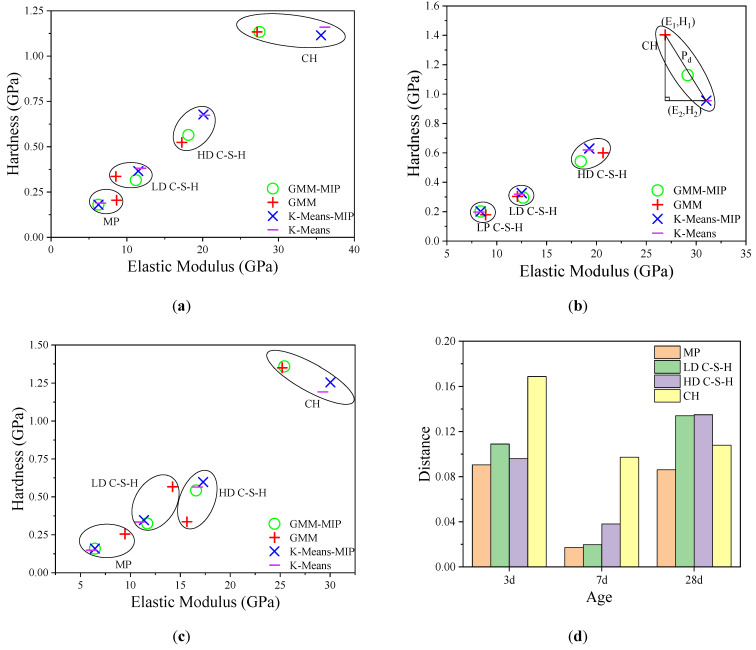
Comparison of Young’s modulus and mean hardness: (**a**–**c**) are 3, 7 and 28 days, respectively; (**d**) is the difference of the mean of each analysis method.

To compare the differences between GMM-MIP, GMM, K-Means-MIP and K-Means. After obtaining the elastic modulus and hardness of each indentation point by using the expressions (1) and (2), the elastic modulus and hardness of different phases are obtained by using the above four methods, and then the results of each phase are averaged, as shown in Figure 7a–c.

It can be seen from the figure that the elastic modulus and hardness of different phases are significantly different. The order from small to large is MP < LD C–S–H < HD C–S–H < CH. MP has the smallest mean elastic modulus and hardness. For example, for 28-day hardened cement paste, the mean values (E, H) obtained by the four methods are K-Means-MIP (6.441 GPa, 0.159 GPa), K-Means (6.080 GPa, 0.149 GPa), GMM-MIP (6.441 GPa, 0.159 GPa), and GMM (9.460 GPa, 0.255 GPa). The mean elastic modulus and hardness of CH are significantly larger than those of the others. There are differences among the results obtained by these four methods. For three-day age cement paste, the calculated results of GMM are close to those of GMM-MIP, and the calculated results of K-Means are close to those of K-Means-MIP. For seven-day hardened cement pastes, except for CH, there is a small gap between the results calculated by different methods. For 28-day-old hardened cement pastes, there is an obvious inconsistency between the results obtained by different methods.

**Figure 8:**
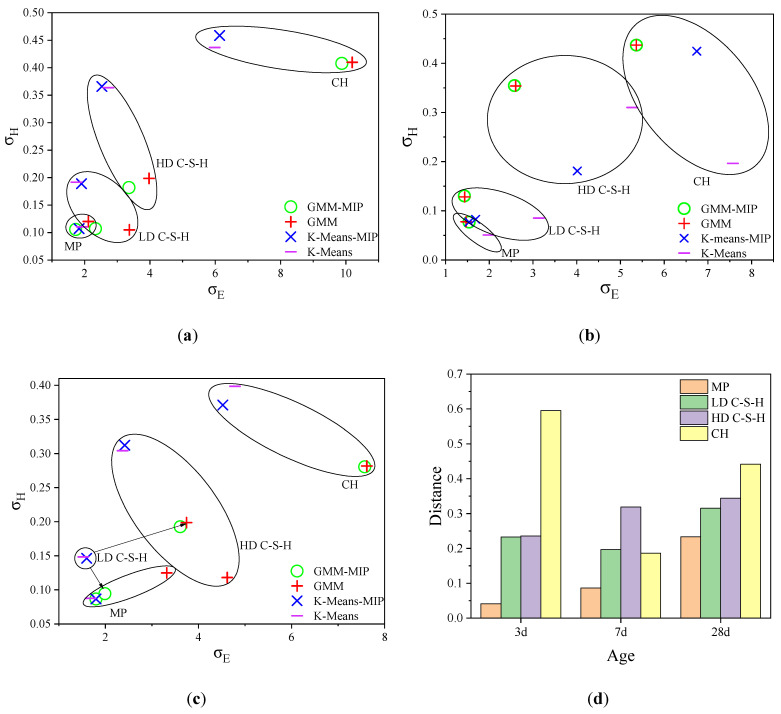
Standard deviation comparison of four methods: (**a**–**c**) standard deviation comparison of 7, 14 and 28 days samples, respectivly; (**d**) differences in standard deviations between analytical methods.

The standard deviation reflects the degree of dispersion of the data. Similar to Figure 7, Figure 8 shows the standard deviation for modulus of elasticity and hardness. From Figure 8a–c, it can be seen that for hardened cement pastes of three ages, the calculation results by the GMM method are generally close to those by the GMM-MIP method. For three-day and 28-day hardened cement pastes, the calculation results of K-Means-MIP and K-means methods are close. However, for seven-day hardened cement pastes, there is a significant difference. This shows that the calculation results based on the GMM method are stable. Figure 8d shows that the CH standard deviation obtained by different methods is more discrete for hardened cement pastes with three-day age and 28-day age. This is mainly due to the significant difference between the results of the K-means method and the GMM method.

**Figure 9:**
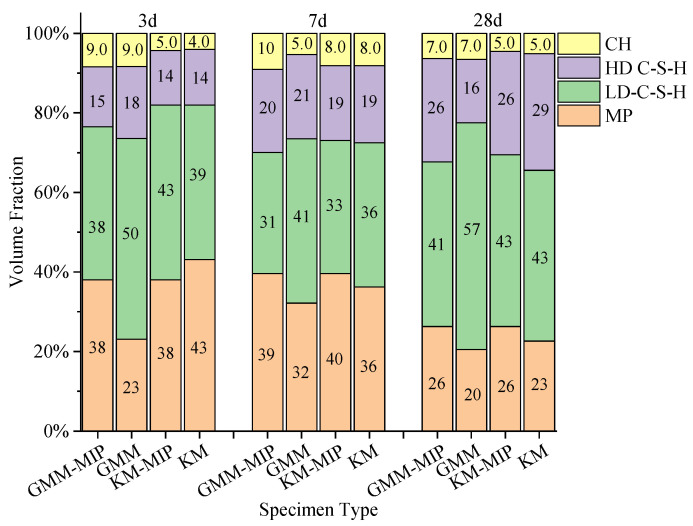
Volume fraction of phases in samples of different ages.

According to the number of indentation points of different phases, the volume fractions of different phases are obtained, as shown in Figure 9.

**Figure 10:**
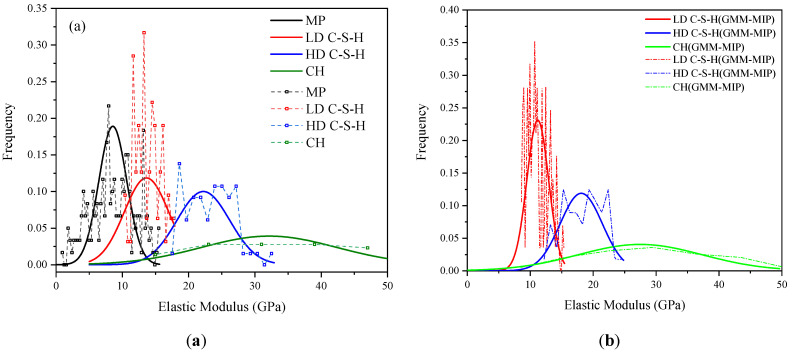
Statistical elastic modulus of each phase at 3-day age: (**a**) statistics of results of phase division by GMM method; (**b**) statistics of results of phase division by GMM-MIP method.

It can be seen from the figure that the volume fractions of each phase vary significantly with age. For the same age, the volume fractions of each phase calculated by different methods differ slightly. For hardened cement pastes of the same age, the calculation results of GMM-MIP, K-means and K-means-MIP methods are close to each other. However, the GMM method differs significantly from their statistical results. This may be because the GMM method assumes that the data points are normally distributed. When the mechanical properties of indentation points do not fully conform to the normal distribution, the solution of the GMM method may be unreasonable. For example, the probability distribution of the elastic modulus is obtained by using the GMM method and GMM-MIP method, respectively, as shown in Figure 10. It can be seen from the figure that the results classified according to GMM differ significantly from the normal distribution, and the results obtained according to GMM-MIP are close to the normal distribution. Therefore, it is suggested to divide MP phase first according to porosity, and then other phases by GMM method, i.e., GMM-MIP method.

### 3.3. Influence of Different Analysis Methods on Phase Division

**Figure 11:**
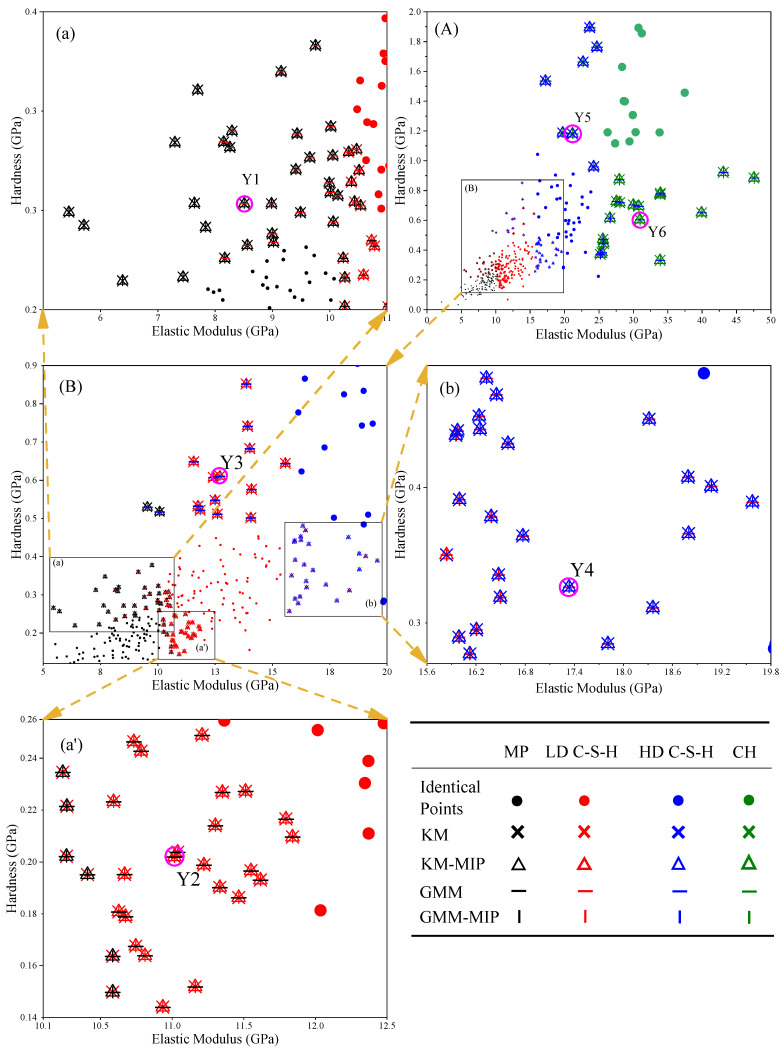
Distribution of elastic modulus and hardness of 3-day-old hardened cement paste, Figures (**a**), (**A**), (**b**), (**a**’) are local enlarged figures of figure (**B**).

**Figure 12:**
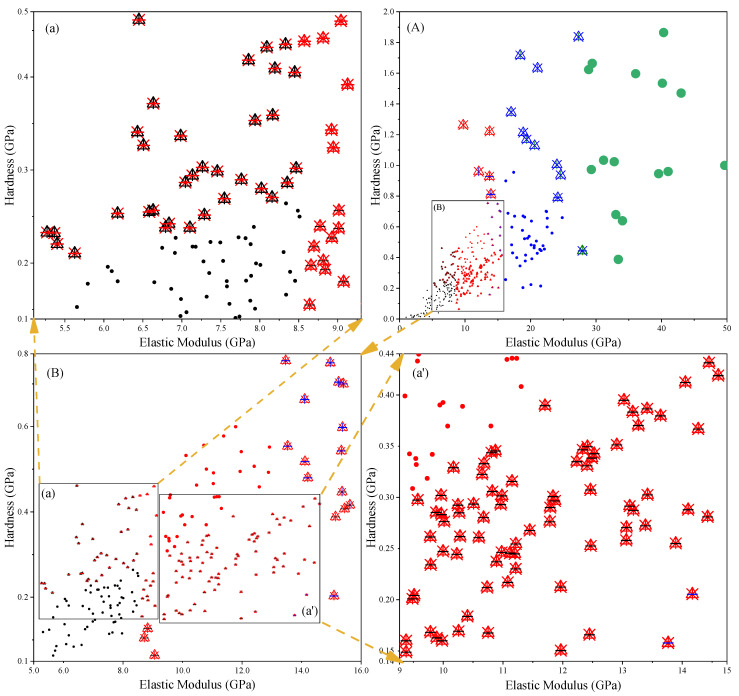
Distribution of elastic modulus and hardness of 7-day-old hardened cement paste, Figures (**a**), (**A**), (**a’**) are local enlarged figures of figure (**B**).

**Figure 13:**
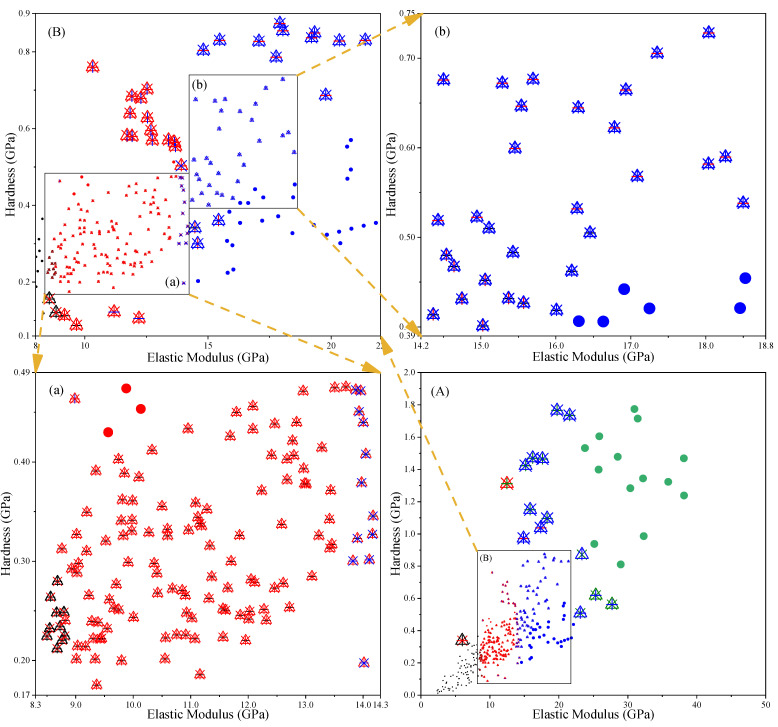
Distribution of elastic modulus and hardness of 28-day-old hardened cement paste, Figures (**a**), (**A**), (**b**) are local enlarged figures of Figure (**B**).

Due to the differences between different algorithms, the same indentation point may be determined as a different phase. Figure 11, Figure 12 and Figure 13 list the phases corresponding to indentation points of different elastic moduli and hardness. The dots in the figure represent the same results obtained by different methods. It can be seen from the diagram that the results obtained by different methods are generally the same, and the differences between the results mainly lie in the intersection position of adjacent phases. This may be because the modulus of elasticity and hardness at these locations are close to each other. Figure 11a shows that partial indentation points are classified as MP by KM and KM-MIP, but these are classified as LD C-S-H phase by GMM. Figure 11 (a’) shows that partial indentation points are classified as MP by GMM-MIP, KM, and KM-MIP, but these are classified as LD C-S-H phase by GMM. Figure 12a shows that in most indentation points, the results of KM-MIP are close to those of GMM-MIP, and the results of KM are close to those of GMM. In a few indentation points, the results of GMM-MIP are inconsistent with those of other methods. Figure 12A shows that the results of the GMM method are different from those of other methods. It can be seen from Figure 13a,b that for most indentation points, the calculation results of GMM are significantly different from those of other methods. The above results show that the results of the GMM method are quite different from those of other methods, and the results of the GMM-MIP method are slightly different from those of KM and KM-MIP. This is because the results of different phases are not the same as those of the normal distribution in the GMM results.

### 3.4. Results of GMM-MIP

**Figure 14:**
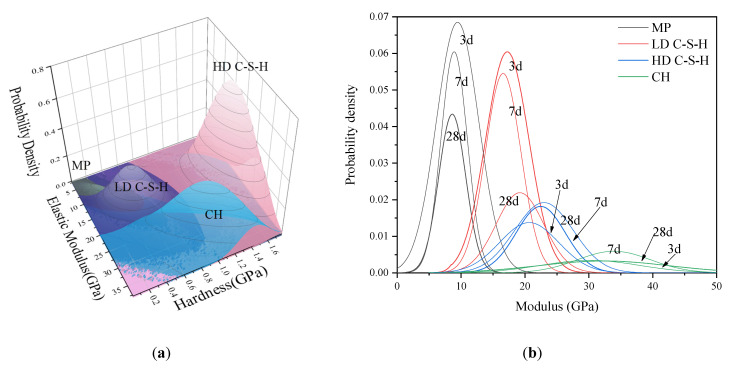
The probability density distribution of elastic modulus and hardness of each phase: (**a**) 28 days sample, (**b**) 3 days, 7 days and 28 days sample.

The comparison between different analysis methods shows that the GMM-MIP method is stable and reliable. Therefore, in this study, the GMM-MIP method was used to analyze the nanoindentation results of hardened cement pastes with different ages, as shown in Figure 14.

Figure 14a shows that the two-dimensional statistical results of elastic modulus and hardness of hardened cement paste at 28 days. Figure 14b shows the statistical results of elastic modulus of hardened cement pastes at different ages. As can be seen from Figure 14a, the change range of elastic modulus and hardness of HD C-S-H is relatively concentrated, while the change range of mechanical properties of the other three phases is relatively scattered. This shows that the mechanical properties of HD C-S-H are different from those of the other three phases, and there are few other three phases mixed in HD C-S-H. This is because MP, LD C-S-H, and C H penetrate each other. As a result, their mechanical properties interact with each other. Through the statistical analysis of all indentation points of 28-day old samples, the elastic modulus and hardness range of different phases are obtained as follows: E(MP) = 6.44 ± 1.80GPa, H(MP) = 0.15 ± 0.09GPa; E(LD C-S-H) = 11.69 ± 1.99 GPa, H(LD C-S-H) = 0.32 ± 0.09 GPa; E(HD C-S-H) = 16.58 ± 3.60 GPa, H(HD C-S-H) = 0.54 ± 0.19 GPa; E(CH) = 25.42 ± 7.57 GPa, H(CH) = 1.35 ± 0.28 GPa.

It can be seen from Figure 14b that the probability density distribution curve of pore phase and LD C–S–H decreases with age, and the coverage of the curve decreases. The probability density distribution curve of CH decreases gradually, but the range increases. With the increase of curing time, hydration products gradually increase, more pore phases are filled, and more LD C–S–H is converted into HD C–S–H.

**Figure 15:**
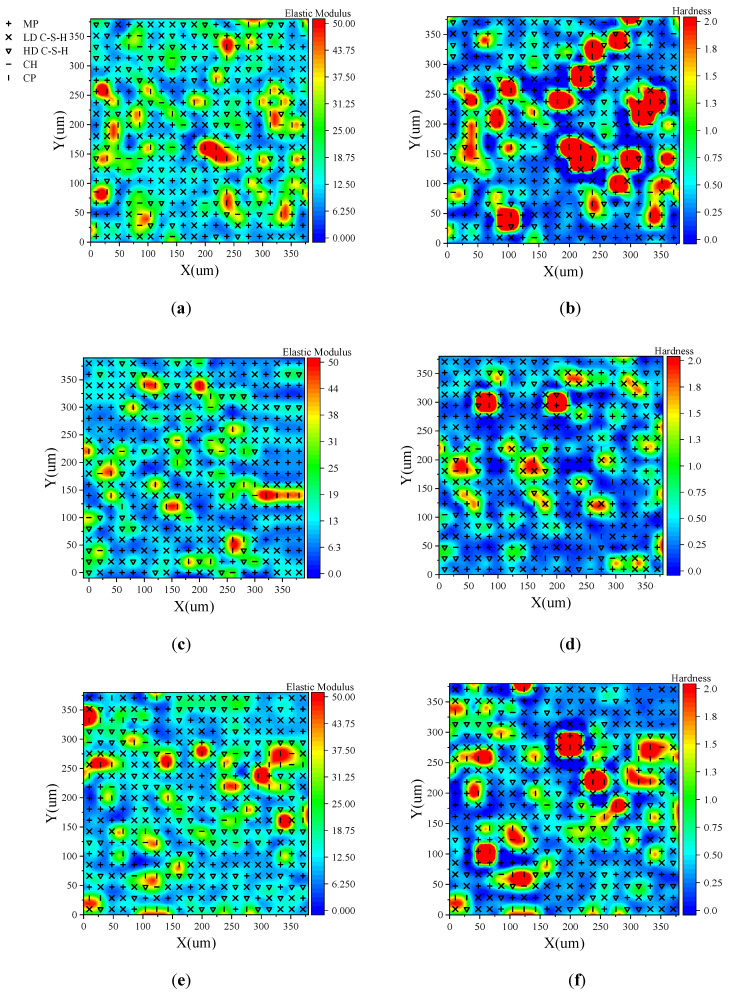
Distribution of elastic modulus and hardness: (**a**) elastic modulus of 3 days specimen, (**b**) 3 days sample hardness, (**c**) elastic modulus of 7 days sample, (**d**) 7 days sample hardness, (**e**) 28 days sample elastic modulus and (**f**) hardness of 28 days sample.

The results of phase division by GMM-MIP method are shown in Figure 15. In this figure, the colour corresponds to the elastic modulus values and hardness for elastic modulus and hardness less than 50 GPa and 2 GPa, respectively. To make the color more visible, the modulus of elasticity > 50 GPa and hardness > 2GPa of all unhydrated cement particles are set to 50 GPa and 2 GPa, respectively. Different symbols indicate the material phase of each indentation point.

It can be seen from the figure that the unhydrated cement particles are generally in the red area. Dark blue areas generally correspond to pore phases. The red is generally surrounded by yellow and green; that is, hydrated calcium silicate and calcium hydroxide around the unhydrated cement particles. This shows that the hydration process of cement particles is from outside to inside. For three-day-old hardened cement paste, the red area and blue area are generally larger. This is because many cement particles do not hydrate in time at an early age, and the elastic modulus and hardness of the cement particles are relatively high, while that of the pore phase around the cement particles are relatively low. The red area for hardness is significantly smaller with increasing age, e.g., for seven-day and 28-day hardened cement pastes. This indicates that more cement particles are involved in the hydration reaction. For hardened cement pastes aged three, seven, and 28 days, the area fractions of LD C–S–H phase and HD C–S–H phase are 52%, 53.5%, and 61.3%, respectively, which indicates that the majority of components in cement particles participate in a hydration reaction and the degree of hydration reaction increases with age.

## 4. Conclusions

In this study, the elastic modulus and hardness of hardened cement paste aged three, seven, and 28 days were tested by nanoindentation. The pore structure of hardened cement paste was obtained by mercury intrusion porosimeter (MIP). A new method for phase division by combined MIP and nanoindentation was proposed, i.e., GMM-MIP and KM-MIP methods. The hydration products are divided into four phases: calcium hydroxide phase (CH), high-density calcium silicate phase (HD C–S–H), low-density calcium silicate phase (LD C–S–H), and pore phase (MP). The main conclusions are as follows:

(1) For the same nanoindentation data, the results of different analysis methods are different. In these results, the mean value of the CH phase is significantly greater than that of the other three phases. However, the dispersion of their mean values is close.
(2) For nanoindentation data where the phases do not completely obey a normal distribution, the content of the individual phases obtained by GMM is different from the remaining three methods. The results obtained for each phase using GMM-MIP, KM-MIP, and KM were relatively close.
(3) The material phase identification method based on the mercury method and nanoindentation indentation is more reasonable than the simple nanoindentation results. According to the GMM-MIP method, the contents of C–S–H in 3 days, 7 days, and 28 days curing age hardened cement paste were 52%, 53.5%, and 61.3%, respectively.
(4) It is recommended that the probability statistical distribution characteristics of the individual material phases are taken into account when identifying the nanoindentation material phases. Only close to the normal distribution of the material phase is suitable for the GMM method. Otherwise, the GMM-MIP, KM-MIP, or KM method is recommended for the material phase identification.

## Figures and Tables

**Table 1:** Chemical composition of cement.

Al_2_O_3_	CaO	Fe_2_O_3_	K_2_O	MgO	Na_2_O	SiO_2_	SO_3_	LOI
6.58	55.30	2.89	0.70	1.52	0.21	23.14	2.56	5.00

## Data Availability

Exclude this statement because the study did not report any data.

## References

[1] Brown L., Allison P.G., Sanchez F. (2018). Use of nanoindentation phase characterization and homogenization to estimate the elastic modulus of heterogeneously decalcified cement pastes. Mater. Des..

[2] Lee H., Vimonsatit V., Chindaprasirt P., Ngo T., Mendis P. (2018). Creep properties of cement and alkali activated fly ash materials using nanoindentation technique. Constr. Build. Mater..

[3] Luo Z.Y., Li W.G., Wang K.J., Shah S.P. (2018). Research progress in advanced nanomechanical characterization of cement-based materials. Cem. Concr. Comp..

[4] Liu J.H., Zeng Q., Xu S.L. (2020). Is scratch test proper to characterize microstructure and mechanical properties of cement-based materials? The effects of loading level and routine. Cem. Concr. Res..

[5] Zhang H.Z., Savija B., Lukovic M., Schlangen E. (2019). Experimentally informed micromechanical modelling of cement paste: An approach coupling X-ray computed tomography and statistical nanoindentation. Compos. Part B Eng..

[6] Němeček J., Lukeš J., Němeček J. (2020). High-speed mechanical mapping of blended cement pastes and its comparison with standard modes of nanoindentation. Mater. Today Commun..

[7] Zhao X.K., Dong Q., Chen X.Q., Fan Q.S., Li R.Q. (2021). Influence of mesoscale heterogeneous and initial defects on the fracture of cement-treated base materials. Constr. Build. Mater..

[8] Gautham S., Sasmal S. (2019). Determination of fracture toughness of nanoscale cement composites using simulated nanoindentation technique. Theor. Appl. Fract. Mech..

[9] Zhang H.Z., Savija B., Schlangen E. (2018). Combined experimental and numerical study on micro-cube indentation splitting test of cement paste. Eng. Fract. Mech..

[10] Akono A.T. (2020). Effect of nano-TiO_2_ on C–S–H phase distribution within Portland cement paste. J. Mater. Sci..

[11] Wilson W., Sorelli L., Tagnit-Hamou A. (2018). Automated coupling of Nanolndentation and Quantitative Energy-Dispersive Spectroscopy (NI-QEDS): A comprehensive method to disclose the micro-chemo-mechanical properties of cement pastes. Cem. Concr. Res..

[12] Wei Y., Gao X., Liang S. (2018). A combined SPM/NI/EDS method to quantify properties of inner and outer C–S–H in OPC and slag-blended cement pastes. Cem. Concr. Comp..

[13] Roa J.J., Phani P.S., Oliver W.C., Llanes L. (2018). Mapping of mechanical properties at microstructural length scale in WC-Co cemented carbides: Assessment of hardness and elastic modulus by means of high speed massive nanoindentation and statistical analysis. Int. J. Refract. Met. Hard Mater..

[14] Li W.G., Kawashima S., Xiao J.Z., Corr D.J., Shi C.J., Shah S.P. (2016). Comparative investigation on nanomechanical properties of hardened cement paste. Mater. Struct..

[15] Krakowiak K.J., Wilson W., James S., Musso S., Ulm F.J. (2015). Inference of the phase-to-mechanical property link via coupled X-ray spectrometry and indentation analysis: Application to cement-based materials. Cem. Concr. Res..

[16] Govender P., Sivakumar V. (2020). Application of k-means and hierarchical clustering techniques for analysis of air pollution: A review (1980–2019). Atmos. Pollut. Res..

[17] Konstantopoulos G., Koumoulos E.P., Charitidis C.A. (2020). Testing Novel Portland Cement Formulations with Carbon Nanotubes and Intrinsic Properties Revelation: Nanoindentation Analysis with Machine Learning on Microstructure Identification. Nanomaterials.

[18] Hou D., Li D., Hua P., Jiang J., Zhang G. (2019). Statistical modelling of compressive strength controlled by porosity and pore size distribution for cementitious materials. Cem. Concr. Comp..

[19] Krakowiak K.J. (2011). Assessment of the Mechanical Microstructure of Masonry Clay Brick by Nanoindentation. Ph.D. Thesis.

[20] Trtik P., Kaufmann J., Volz U. (2012). On the use of peak-force tapping atomic force microscopy for quantification of the local elastic modulus in hardened cement paste. Cem. Concr. Res..

[21] Marteau J., Jourani A., Bigerelle M. (2020). Determination of an Objective Criterion for the Assessment of the Feasibility of an Instrumented Indentation Test on Rough Surfaces. Materials.

[22] Oliver W., Pharr G. (1992). An improved technique for determining hardness and elastic-modulus using load and displacement sensing indentation experiments. J. Mater. Res..

[23] Zhang M.H., Li H. (2011). Pore structure and chloride permeability of concrete containing nano-particles for pavement. Constr. Build. Mater..

[24] Yang C.C. (2006). On the relationship between pore structure and chloride diffusivity from accelerated chloride migration test in cement-based materials. Cem. Concr. Res..

[25] Ying J., Zhou B., Xiao J. (2017). Pore structure and chloride diffusivity of recycled aggregate concrete with nano-SiO_2_ and nano-TiO_2_. Constr. Build. Mater..

[26] Ulm F., Vandamme M., Bobko C.P., Ortega J.A., Tai K., Ortiz C. (2007). Statistical indentation techniques for hydrated nanocomposites: Concrete, bone, and shale. J. Am. Ceram. Soc..

